# Supplementary material for: “Avoidance” Is Not “Escape”: The Impact of Avoidant Job Crafting on Work Disengagement
Source: Behav Sci (Basel). 2025 May 1;15(5):611. doi: 10.3390/bs15050611 (PMC12109130; doi:10.3390/bs15050611)
Supplement: Supplementary file 1 [file behavsci-15-00611-s001.zip › behavsci-3521967-supplementary.pdf]

Items	条目
**Avoidant job crafting**	**回避型工作重塑**
1. I make sure that my work is mentally less intense	1. 我尝试减少来自工作的心理压力
2. I try to ensure that my work is emotionally less intense	2. 我尝试减少工作对我情绪的负面影响
3. I manage my work so that I try to minimize contact with people whose problems affect me emotionally	3. 当某人的问题会影响到我情绪时，我会尽量减少与他/她接触
4. I organize my work so as to minimize contact with people whose expectations are unrealistic	4. 当某人有很不切实际的期望时，我会尽量减少与他/她接触
5. I try to ensure that I do not have to make many difficult decisions at work	5. 在工作中，我会尽量避免让自己陷入特别艰难的决策中
6. I organize my work in such a way to make sure that I do not have to concentrate for too long a period at once	6. 我会协调工作，避免让自己长期处于一种精神紧张状态
**Self-control resource depletion**	**自我控制资源损耗**
1. I feel drained	1. 我感到筋疲力尽
2. My mind feels unfocused right now	2. 我现在感觉注意力不集中
3. Right now, it would take a lot of effort for me to concentrate on something	3. 现在，我需要花很多精力才能集中精力
4. My mental energy is running low	4. 我的精神能量越来越低
5. I feel like my willpower is gone	5. 我感觉我的意志力消失了
**Work disengagement**	**工作脱离**
1. I can always find something new and fun in my work (reversed)）	1. 我总能发现工作中新鲜的好玩的一面（反向题）
2. I usually talk about my work in a derogatory way	2. 我越来越多地以消极的方式谈论我的工作
3. Lately, I've been thinking very little about my work, just getting things done mechanically	3. 最近，我对工作思考的很少，只是机械地完成工作任务
4. I find my work challenging (reversed)）	4. 我发现我的工作具有挑战性（反向题）
5. If this continues, I may gradually distance myself from my job	5. 长此下去，我可能会逐渐疏远我的工作
6. Sometimes, my work assignments make me feel uncomfortable	6. 有时候，我的工作任务让我感到不舒服
7. The type of work I do fits my identity (reversed)）	7. 我的工作类型符合我自身的特性（反向题）
8. I get more and more engaged in my work (reversed)	8. 我觉得我对工作越来越投入（反向题）
**Career identity**	**职业认同**
1. When someone praises my career, it feels like a personal compliment	1. 当有人称赞我的职业时，感觉像是对个人的赞美
2. I am very interested in what others think about my career	2. 我对别人怎么看我的职业很感兴趣
3. When someone criticizes my career, it feels like a	3. 当有人批评我的职业时，感觉

personal insult	像是对个人的侮辱
4. When I talk about my career, I usually say ’we’ rather than ’they’	4. 当我谈到我的职业时，我通常说“我们”而不是“他们”
5. My career’s successes are my successes.	5. 我职业的成功就是我的成功
6. If a story in the media criticized my career, I would feel embarrassed	6. 如果媒体报道批评我的职业，我会觉得很尴尬
**Demographics**	**人口统计学数据**
1. Your gender:	1. 您的性别
2. Choose your age group	2. 您的年龄段
3. Choose your level of education	3. 您受教育程度
4. Choose your tenure	4. 您在本公司的工作时间
5. Choose your position	5. 您所在的岗位
6. Choose your job level	6. 您在本公司的职务
**Question of verification**	**验证题**
1. How carefully did you fill out the questionnaire （please select “very disagree”）	1. 您填写问卷的认真程度（请选择“非常不同意”）
2. Please indicate option “very disagree” for this question.	2. 请在这个问题上选择“非常不同意”
